# An Open-Source and Highly Adaptable Rodent Limited Bedding and Nesting Apparatus for Chronic Early Life Stress

**DOI:** 10.1523/ENEURO.0081-25.2025

**Published:** 2025-06-19

**Authors:** Olivia S. O’Neill, Dylan J. Terstege, Amisha K. Gill, Moriah Edge-Partington, Raksha Ramkumar, Jonathan R. Epp, Derya Sargin

**Affiliations:** ^1^Department of Psychology, University of Calgary, Calgary, Alberta T2N 1N4, Canada; ^2^Hotchkiss Brain Institute, University of Calgary, Calgary, Alberta T2N 4N1, Canada; ^3^Alberta Children’s Hospital Research Institute, University of Calgary, Calgary, Alberta T2N 4N1, Canada; ^4^Department of Cell Biology and Anatomy, University of Calgary, Calgary, Alberta T2N 4N1, Canada; ^5^Cumming School of Medicine, University of Calgary, Calgary, Alberta T2N 4N1, Canada; ^6^Department of Physiology and Pharmacology, University of Calgary, Calgary, Alberta T2N 4N1, Canada

**Keywords:** customizable laboratory equipment, early life stress, fragmented maternal care, limited bedding and nesting, mouse models, open source

## Abstract

Early life stress (ELS) increases susceptibility to cognitive and socioemotional dysfunction by disrupting the neurobiological systems that regulate these behaviors. Animal models provide a valuable tool for investigating the underlying mechanisms, enabling precise manipulation of stress exposure during development. The limited bedding and nesting (LBN) model, which induces maternal stress by restricting access to bedding and nesting materials in rodents, has been instrumental in advancing our understanding of chronic ELS. While this paradigm has been widely adopted, variations in apparatus designs and subtle differences in methodologies could impact consistency across studies. Here, we provide standardized guidelines for a cost-effective open–source mouse LBN apparatus design, which could further enhance the model's utility while supporting pup survival. We additionally present our findings observed during the duration of the LBN paradigm, which spans from postnatal day (PND) 2 to 10, for both dams and pups. We observe comparable corticosterone in control and LBN dams from PND 3 to 5. However, from PND 6 to 10, corticosterone remains elevated in LBN dams, while control dams show a decline. Notably, the LBN paradigm disrupts maternal care, as LBN dams exhibit more frequent nest exits and stereotypic behaviors during the dark phase. At PND 10, pups exhibit significantly reduced blood serum corticosterone levels and lower body weight compared with those reared under control conditions. By providing open-source equipment and detailed experimental protocols, our work aims to build on existing LBN paradigms to further enhance the accessibility and reproducibility of chronic ELS models.

## Significance Statement

Early life stress (ELS) paradigms have been widely adapted and instrumental in establishing various critical influences for studying the mechanisms of developmental stress. Building on the previously established models, this manuscript outlines a detailed protocol to construct an open-source and cost-effective limited bedding and nesting paradigm to induce chronic ELS in rodent models. This 3D-printed cage and platform design is easily implementable across various laboratories, featuring temperature control to support pup survival and an overhead camera for continuous behavioral recording during stress manipulation. The highly adaptable and customizable design promotes experimental transparency and strengthens the translational value of future findings derived from ELS models.

## Introduction

Neural circuitry is highly malleable during development, rendering it susceptible to alterations. Under adverse circumstances however, this malleability can lead to maladaptive outcomes. ELS is associated with changes in brain function that can have lasting effects on cognitive performance and emotional behavior. Through alterations in hypothalamic–pituitary–adrenal (HPA) axis reactivity (McEwen, 1998; Ganella et al., 2015), neural connectivity (Bravo et al., 2014; Kooiker et al., 2024; Ramkumar et al., 2024), and overall brain activity (Kopala-Sibley et al., 2020); ELS has been suggested to increase the risk of affective disorders (Cicchetti and Curtis, 2005; Dillon et al., 2009; Hu et al., 2020; Menezes et al., 2024). However, ELS spans a broad spectrum of stressors, including family conflict, neglect, parental stress, maltreatment, and poverty-related challenges. Epidemiological evidence suggests that despite the diverse range of stressors, the association between ELS and increased risk of psychopathology remains robust (Kessler et al., 2010; Essex et al., 2011). However, the mechanisms underlying this association may vary across various types of ELS. Therefore, establishing rodent models of ELS stressors is crucial for investigating the underlying mechanisms in detail.

One widely used and adopted rodent model of ELS is the limited bedding and nesting (LBN) paradigm, developed by Baram and colleagues (Ivy et al., 2008; Rice et al., 2008; Walker et al., 2017). The LBN paradigm simulates a low-resource environment by restricting the dam's access to bedding and nesting materials during a critical developmental window [postnatal day (PND) 2–9; Ivy et al., 2008; Rice et al., 2008]. Limited access to nesting materials creates a stressful environment, altering the dam's interactions with the pups (Orso et al., 2019), with consequences for cognitive function (Naninck et al., 2015), anxiety regulation (Dalle Molle et al., 2012), and depressive-like behaviors (Goodwill et al., 2019). While the LBN model is increasingly utilized, the apparatus designs employed to create this stressful environment could vary across studies. The variability, as comprehensively reviewed by Walker et al. (2017), may contribute to differences in outcomes, making cross-study comparisons more challenging. To enhance our understanding of the mechanisms underlying the effects of LBN-induced stress, adopting standardized protocols could be beneficial.

Here, we introduce an open-source design for LBN platforms and cages, which enables the integration of overhead cameras to monitor maternal behavior across both light and dark phases. We also incorporate a temperature control feature to mitigate hypothermia-related pup loss, which can stem from varying environmental conditions across animal facilities. Under these conditions, we report our observations on stress-related alterations in dams and pups during the LBN paradigm.

## Materials and Methods

### LBN cage and platform construction

**Figure 1.**
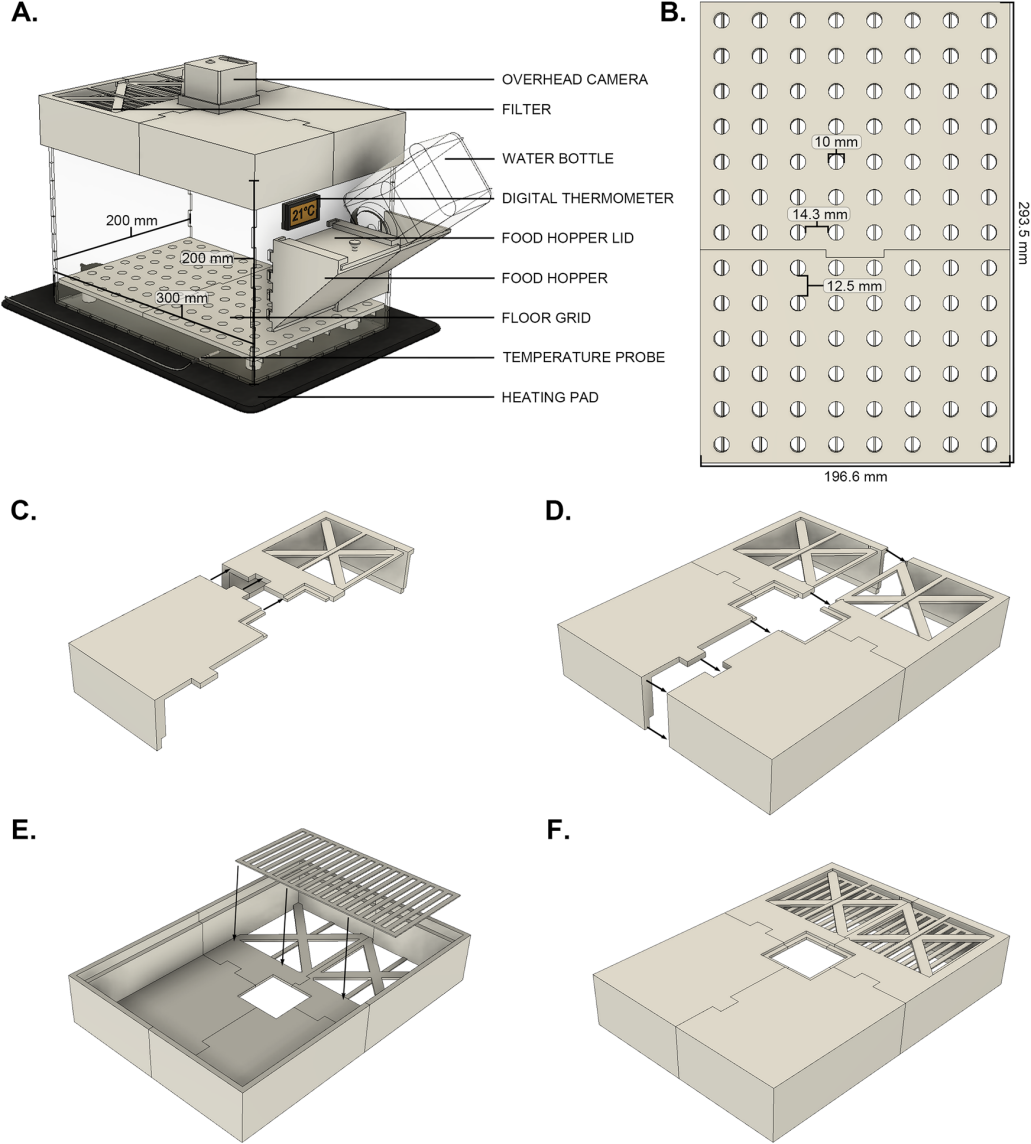
LBN cage design. ***A***, Model of the LBN cage, with all components labeled. Cage dimensions are provided. ***B***, Schematic of the LBN cage floor grid, illustrating the diameter of the holes which form the grid and the spacing between. During the assembly of the LBN cage lid, it is recommended that users (***C***) construct the lid one half at a time (LBN_lid_fm.stl with LBN_lid_mm.stl and LBN_lid_ff.stl with LBN_lid_mf.stl) by applying a thin layer of acetone to the surfaces where components will touch. These components should be held together firmly as the bond cures. After having assembled both halves of the lid, (***D***) these components can be joined, with acetone being applied on common surfaces. Components should be held together firmly during this process. Finally (***E***) on the underside of the lid, the grate-like component (LBN_lid_grate.stl) is adhered to the lid using acetone. ***F***, Completed cage lid.

All components for the LBN and control cages were designed using Autodesk Fusion 360 (Version 2.0.20981). All 3D-printed components were printed using a Creality Ender 3 V2 3D printer equipped with a frosted glass build plate. The STL files were sliced using Ultimaker Cura (v4.8.9). For all prints, the 1.75 mm PLA material was extruded through a 0.6 mm brass nozzle at 40 mm/s with a layer height of 0.28 mm. The components were printed with an infill of 20%. These settings represent relatively accessible printing setups with conservative manufacturing times; however, these may vary depending on the printer and the materials being used. In total, with these settings, 5 d were required to produce each cage. It is likely that other manufacturing setups will be able to reliably accommodate printing these components at higher speeds. Please see Figure 1 for a detailed schematic of the cage and platform components.

#### Cages

The 0.25 in transparent plexiglass sheets were obtained using a laser cutter (Boss Laser, LS-2440 CO2 Laser Cutter and Engraver). The interlocking teeth of the sheets were bonded together using acrylic solvent (Weld-On #3 Acrylic Cement, SKU 160145) and allowed to cure for 24 h. Once fully assembled, the cages have dimensions of 300 × 205 × 200 mm (length × width × height).

#### Food and water bottle holder

The food and water bottle holder components (LBN_hopper_body.stl and LBN_hopper_lid.stl) were printed over the course of a total of 8 h, using the previously described conservative 3D printer settings. After printing, metal bars (100 × 2 mm stainless steel round rods) were vertically affixed to the front of the food hopper using hot glue. The food hopper and water bottle holder unit were then adhered to the cage with acrylic solvent (Weld-On #3 Acrylic Cement, SKU 160145) and allowed to cure for 24 h.

#### Cage lid

Working within the constraining dimensions of the Creality Ender 3 V2 printer bed, the cage lid was printed as multiple interlocking components. The four main components (LBN_lid_mm.stl, LBN_lid_mf.stl, LBN_lid_fm.stl, and LBN_lid_ff.stl) were each printed in 4 h. Cage lid assembly has been depicted in Figure 1*C*–*F*. These pieces were joined by carefully applying a thin layer of superglue and accelerant along the connecting surfaces and firmly holding the components together. Afterward, a thin grate-like component (LBN_lid_grate.stl) was printed in 30 min and adhered within the opening in the lid. This grate prevents mice from chewing through a piece of filter paper, which was inserted in the space above the grate. Finally, a removable cover was printed for the upright camera hole in the center of the lid. This lid was used to close the hole while home-cage behavior was not being recorded.

#### LBN platform

A floor insert with holes was designed to prevent the accumulation of urine and feces in the absence of bedding materials. The 299 × 304 mm insert has 104 holes (10 mm diameter) arranged in an 8 × 13 grid. To prevent the pups from falling through, a crossbeam was affixed across the bottom of each hole. Working within the constraining dimensions of the Creality Ender 3 V2 printer bed, the floor grid was printed as two interlocking components (LBN_grid_m.stl and LBN_grid_f.stl). The floor grid was printed with 20 mm legs to keep it elevated from the floor of the cage. This allowed for a thin layer of bedding to be laid down underneath the platform and out of reach from the dams. Each of these components required 6 h of printing time using the previously described conservative printer settings. After printing, these components were assembled using superglue and a thin layer of accelerant and then left to cure for 24 h. The floor grids were used exclusively in the LBN cages and omitted in the control cages.

### Animals

Male and female C57BL6/J mice bred in house were used for all experiments. Mice were housed under a 12 h light/dark (light on at 07:00; zeitgeber time, 00:00) with *ad libitum* access to food and water. All experimental procedures were performed in accordance with the guidelines established by the Canadian Council on Animal Care and were approved by the Life and Environmental Sciences Animal Care Committee at the University of Calgary.

### LBN protocol

Following cage assembly, a thin layer of absorbent (∼0.5 cm layer covering the entire cage bottom), woodchip, bedding materials (laboratory grade aspen chip, Northeastern Products) were used to sparingly cover the cage floor underneath the perforated platform. This layer of bedding was used to appropriately manage the odor of urine and feces. On PND 2, dams and pups were moved to the custom LBN cages and placed on top of the platform. Dams were provided with half of a single cotton nestlet. Cages were maintained at a temperature of 21°C from PND 2 to 4 using a subcage heater composed of a heating pad with an attached digital thermostat (obtained from Amazon) to monitor the temperature of the cage. The thermostat probe was inserted into the bedding through a small hole opened near the bottom of the back side of the cage. Importantly, the hole was positioned below the LBN platform, to prevent the dams from chewing on the probe cable. On PND 10, all their pups were killed for blood serum collection.

### Maternal behavior

Home-cage behavior was recorded using a camera (Wyze Cam v3) mounted to the lid of each cage. Recording was conducted continuously throughout the duration of the LBN paradigm. Maternal behaviors were assessed manually, scoring nest entries, and the time spent in and outside the nest for a 1 h period during the light and dark (2:00–3:00) phases on PNDs 4, 6, and 9. Tail-chasing behavior observed in LBN dams was quantified by counting the number of rotations during the same time period.

### Enzyme linked immunosorbent assay (ELISA) for corticosterone quantification

Fecal samples were collected daily from control and LBN dams from PND 3 to 10. The samples were pooled for processing and analysis into three time periods: PNDs 3–5, 6–8, and 9–10. The fecal samples were stored at −80°C and then dried at 40°C overnight and grounded into a powder prior to assay preparation. Corticosterone was extracted according to the manufacturer's protocol (DetectX Steroid Solid Extraction, Arbor Assays). Accordingly, dried and powdered samples were treated with ethanol (1 ml/0.1 g fecal matter) for 1 h on a shaker and then centrifuged at 4°C, 5,000 rpm. The supernatant was evaporated to isolate the sample extract. The concentration (pg/ml) of corticosterone was measured according to the manufacturer's instructions (Arbor Assays, DetectX Corticosterone Kit, K014). Absorbance at 450 nm was measured using the BioLegend Mini ELISA plate reader (BioLegend).

At PND 10, pups were sacrificed for blood serum collection. Trunk blood samples were stored at 4°C overnight to allow clotting and then centrifuged at 10,000 rpm for 15 min. The separated supernatant (serum) was stored at −20°C until assay (Eid et al., 2019). Corticosterone concentration (pg/ml) was measured according to the manufacturer's instructions (Arbor Assays, DetectX Corticosterone Kit, K014).

### Statistical analysis

Statistical analyses were conducted using Prism (version 9.1.0, GraphPad Software). Data were analyzed using two-way ANOVA, unpaired *t* test, Mann–Whitney *U* test, and linear regression analysis. All data were presented as mean ± standard error of the mean (SEM). When appropriate, post hoc analysis was performed using Bonferroni's multiple-comparison test. Statistical results are presented in Extended Data Figure 2-1.

### Data availability

Any data generated during the current study will be made available online at https://osf.io/krhsz/?view_only=13ad724f054b4ba0ae5c8c006f4eb145.

## Results

### Dams show elevated corticosterone levels during the LBN paradigm

**Figure 2.**
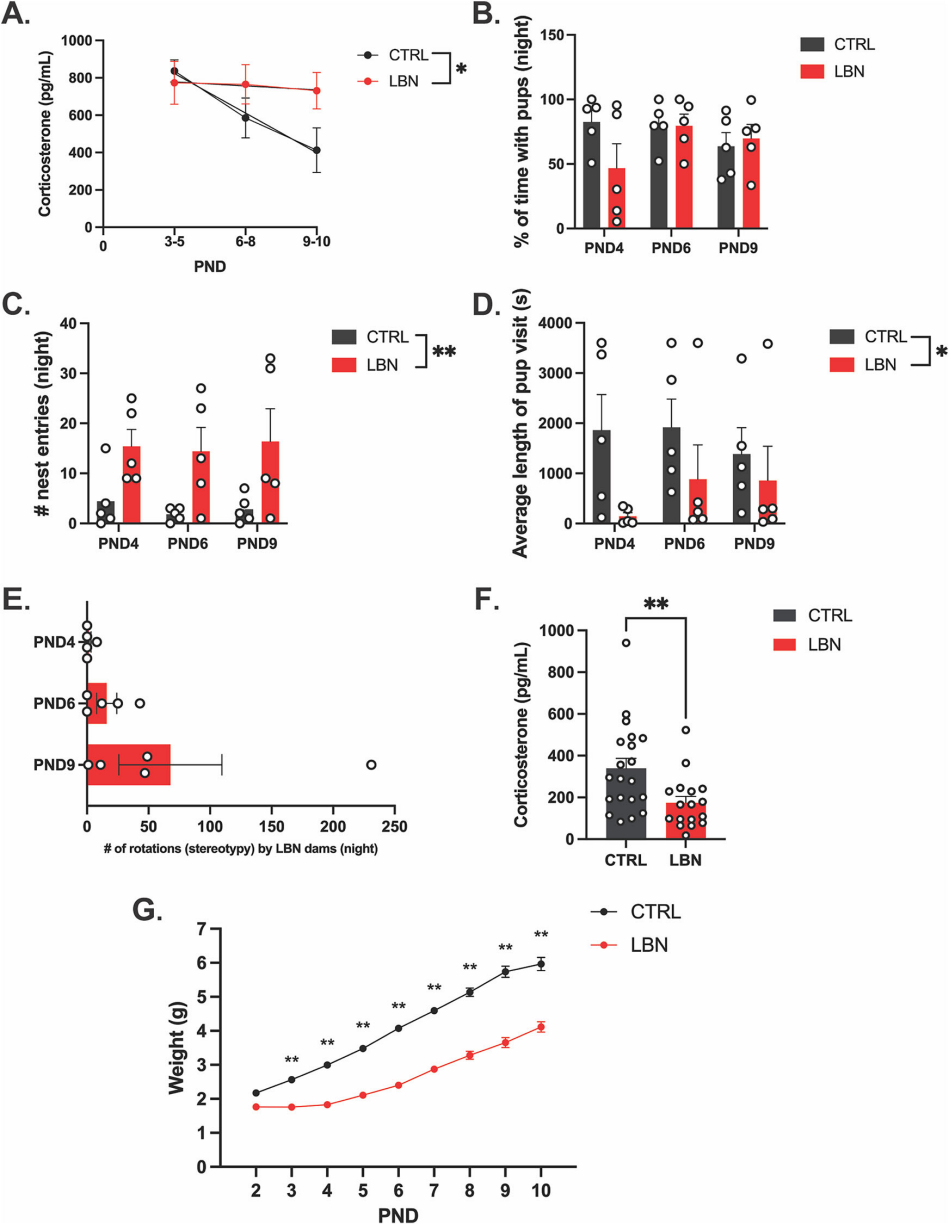
The LBN model is sufficient to promote stress and altered maternal behaviors in dams, leading to alterations in pup stress response and decreased pup weights. ***A***, Fecal corticosterone levels from control and LBN dams pooled and measured for PNDs 3–5, 6–8, and 9–10. LBN dams (*n* = 3) maintained high levels of corticosterone in fecal samples over the duration of the LBN paradigm (*R*^2^ = 0.01345; *F*_(1,7)_ = 0.09545; *p* = 0.7664) compared with controls (*n* = 3; *R*^2^ = 0.6014; *F*_(1,7)_ = 10.56; *p* = 0.0141; nonlinear fit, *F*_(2,14)_ = 3.743; *p* = 0.0499). By PND 9–10, LBN dams had significantly higher levels of fecal corticosterone than control dams (*t*_(12)_ = 2.196; *p* = 0.0484). ***B***, The percentage of time spent with pups during the dark phase (2:00–3:00) on PNDs 4, 6, and 9. LBN (*n* = 5) and control dams (*n* = 5) spent comparable time with pups during the dark phase (2:00–3:00) on PNDs 4, 6, and 9 (two-way ANOVA interaction, *F*_(2,24)_ = 1.874; *p* = 0.1754). ***C***, The number of nest entries by control (*n* = 5) and LBN dams (*n* = 5) during the dark phase (2:00–3:00) on PNDs 4, 6, and 9. LBN dams show a greater number of nest exits than controls across time points, suggesting more fragmented bouts of maternal care (two-way ANOVA effect of housing condition, *F*_(1,24)_ *=* 16.07; *p* = 0.0005). ***D***, Average duration of visits to the nest during the dark phase (2:00–3:00) on PNDs 4, 6, and 9. LBN dams (*n* = 5) demonstrated a reduction in the average length of nest visit compared with controls (*n* = 5; two-way ANOVA effect of housing condition, *F*_(1,24)_ = 5.269; *p* = 0.0307). ***E***, The number of tail-chasing and twirling stereotypical rotations observed in LBN dams during the dark phase (2:00–3:00) on PNDs 4, 6, and 9. ***F***, Blood serum corticosterone concentration in LBN and control pups measured from PND 10. LBN pups show a significant reduction in serum corticosterone concentration than controls [LBN (*n* = 17) vs control (*n* = 20); Mann–Whitney *U* test, *U* = 78; *p* = 0.0044]. ***G***, Average body weight of LBN (*n* = 17) and control pups (*n* = 20) over the duration of the LBN paradigm. We observed significantly lower body weight in LBN pups compared with controls from PND 3 to 10 (two-way ANOVA interaction, *F*_(8,315)_ = 13.48; *p* < 0.0001; post hoc LBN vs CTRL, PND 2, *p* = 0.0632; PNDs > 2; *p* < 0.0001). Please see Extended Data Figure 2-1 for statistical analysis presented in this figure. Data represent mean ± SEM; *p < 0.05; **p < 0.01; CTRL, control; LBN, limited bedding and nesting; PND, postnatal day.

Corticosterone levels were assessed throughout the duration of the protocol by collecting fecal samples from control and LBN dams on PNDs 3–10. Samples collected on PNDs 3–5, 6–8, and 9–10 were pooled, and corticosterone levels were measured across three time points. Levels were comparable between control and LBN dams on PNDs 3–5. Parturition represents a stressful event for dams, so we expected elevated stress hormone levels in both control and LBN mice in the early postpartum period but a different relationship between postpartum time and corticosterone levels in each group. To model these relationships, we performed linear regression analysis. Our results revealed that corticosterone levels in control dams significantly decreased over time, suggesting an adaptation to postpartum conditions (*F*_(1,7)_ = 10.56; *p* = 0.0141; *R*^2^ = 0.6014). In contrast, corticosterone levels in LBN dams remained chronically elevated throughout the entire duration of the LBN paradigm (*F*_(1,7)_ = 0.09545; *p* = 0.7664; *R*^2^ = 0.01345), suggesting heightened stress levels under LBN conditions (Fig. 2*A*). A statistical comparison of fits indicated a significant difference between control and LBN groups in the change of corticosterone levels over time (*F*_(2,14)_ = 3.74; *p* = 0.049). We also performed a two-way ANOVA to compare corticosterone levels of control and LBN dams across the stress period. We observed a nonsignificant trend between groups (*F*_(1,12)_ = 3.020; *p* = 0.108). However, as we expected the corticosterone effect size to be the largest at the latest time point, we performed planned comparisons between control and LBN corticosterone levels at PNDs 9–10. Fisher's LSD revealed that control dams had significantly lower fecal corticosterone than LBN dams at PNDs 9–10 (*t*_(12)_ = 2.196; *p* = 0.0484). Overall, persistent elevated corticosterone levels in LBN dams highlight the prolonged stress induced by the LBN paradigm.

10.1523/ENEURO.0081-25.2025.f2-1 Figure 2-1 Extended statistical results supporting Figure 2. Download Figure 2-1, DOC file.

### LBN induces abnormal maternal behavior

LBN has consistently been shown to alter maternal care, leading to fragmented, dysfunctional nurturing behaviors in dams (Ivy et al., 2008; Rice et al., 2008; Bath et al., 2016; Molet et al., 2016; Moussaoui et al., 2016; Gallo et al., 2019; Ramkumar et al., 2024), which could be a critical factor underlying dysregulations in emotional and cognitive development (Baram et al., 2012; Molet et al., 2016). To validate these findings with the modified LBN paradigm, we observed and quantified maternal behaviors in control and LBN dams, focusing on the time spent with pups and the frequency of nest entries and exits. We quantified the dam behavior during the active/dark phase (2:00–3:00 A.M.) at the beginning (PND 4), middle (PND 6), and end (PND 9) of the LBN paradigm. For the duration of the paradigm, LBN and control dams spent a comparable time interacting with pups in the nest (*F*_(2,24)_ = 0.06; *p* = 0.9420; Fig. 2*B*). During the dark phase, LBN dams entered the nest significantly more frequently for pup interactions compared with control dams (*F*_(1,24)_ = 16.07; *p* = 0.0005; Fig. 2*C*). LBN dams had significantly shorter average length of pup/nest visits compared with controls (*F*_(1,24)_ = 5.269; *p* = 0.0307; Fig. 2*D*). Additionally, we observed frequent tail-chasing and twirling stereotypical behaviors in LBN dams during the dark phase, which were completely absent in control dams (Fig. 2*E*). Altogether, our data showed that the modified LBN paradigm results in fragmented maternal care, which is consistent with changes reported in previous studies (Ivy et al., 2008; Rice et al., 2008; Bath et al., 2016; Molet et al., 2016; Gallo et al., 2019). Additionally, we report for the first time the presence of stereotypical behaviors performed to varying degrees exhibited exclusively by LBN dams during the dark phase. While stereotypies may not be direct indicators of stress, they are commonly observed in animals exposed to barren or resource-limited environments (Cooper and Nicol, 1991; Gross et al., 2012).

### LBN induces reduced corticosterone levels and decreased body weight in pups

On PND 10, trunk blood was collected from control and LBN pups to measure serum corticosterone concentration. This revealed significantly lower corticosterone levels in serum obtained from LBN pups compared with the controls [LBN (*n* = 17) versus control (*n* = 20), Mann–Whitney *U* test: *U* = 78; *p* = 0.0044; *p* = 0.004; Fig. 2*F*]. LBN-induced reduction in corticosterone levels in pups has previously been reported (Moussaoui et al., 2016); however, other studies found increased corticosterone levels in pups following LBN (Rice et al., 2008). While these observations suggest altered stress regulation, reduced corticosterone levels may result from an ELS-induced delay in stress response maturation (Heim et al., 2000; Kaufman et al., 2000; Elzinga et al., 2008; van Bodegom et al., 2017).

The body weight of pups was recorded daily from PND 2 to 10. Although LBN pups gained weight over time, the body weights remained significantly lower compared with the controls (two-way ANOVA; *F*_(8,315)_ = 13.48; *p* < 0.0001; Fig. 2*G*). Post hoc analyses revealed significantly lower body weight of pups reared under LBN conditions compared with controls from PND 3 to 10 [all *p*'s < 0.0001; mean weight gain ± SEM, LBN, 2.155 ± 0.138; CTRL, 3.793 ± 0.175; LBN (*n* = 23); CTRL (*n* = 20); Mann–Whitney *U* test, *U* = 26; *p* < 0.0001]. These findings are consistent with previous studies (Rice et al., 2008; Arp et al., 2016). Pup mortality may be observed in ELS paradigms. While our model does not eliminate this outcome, we only see a 7.15% drop in survival between our control pups (*n* = 25; 100% survival) and LBN pups (*n* = 28; 92.85% survival).

For detailed statistical results, see Extended Data Figure 2-1.

## Discussion

Here, we provide instructions for constructing an open-source LBN apparatus for mice to study chronic ELS induced by resource scarcity. Using this modified paradigm, we found persistently elevated corticosterone in LBN dams and reduced plasma corticosterone in LBN-reared pups at PND 10, compared with controls. As reported previously, we observed fragmented maternal care without significant reduction in rearing time, a pattern linked to various developmental consequences (Ivy et al., 2008; Rice et al., 2008; Molet et al., 2016). Building on the foundational work of the previously developed LBN conditions (Ivy et al., 2008; Rice et al., 2008), our design provides an accessible and cost-effective blueprint for studying chronic ELS, to further advance our understanding of ELS.

The design uses accessible laboratory materials and equipment, making the LBN model easy to implement across various settings. Customizable floor grid and cage components allow adaptation to various cage sizes, species needs, animal care guidelines, and space constraints. The grid design, similar to the previous wire mesh platforms, minimizes feces and urine accumulation (Walker et al., 2017). However, researchers should still monitor for urine-derived ammonia exposure and ensure proper ventilation in rodent colony rooms. A potential limitation of the LBN paradigm is increased pup mortality. While this risk is not entirely eliminated, our design includes features that prioritize pup survival while maintaining the ELS phenotype. The grid design improves comfort over mesh floors, potentially mitigating animal welfare concerns and enhancing pup survival. To prevent hypothermia-related pup mortality, a thermostat and subcage heater were used to maintain the cage temperature at 21°C from PND 2 to 4.

To evaluate the effects of the modified LBN apparatus on dams and pups, we quantified key parameters commonly reported in LBN paradigms. In line with previous studies (Ivy et al., 2008; Moussaoui et al., 2016), our model appears to produce erratic, fragmented maternal care in LBN dams, characterized by an increased nest entries and exits, along with shorter pup interactions. This outcome has been critically linked to ELS and impaired emotional and cognitive development in both humans and rodent models (Heim et al., 2000; Heim and Nemeroff, 2001; Molet et al., 2016). ELS induced by lower-quality maternal care, rather than maternal separation, may better model chronic developmental stress in humans, where maternal presence remains but external factors compromise the quality of care (Walker et al., 2017). To facilitate effective recording and analysis of these alterations, the LBN cage lids were designed with a dedicated space for mounting an overhead camera and an attachable cover for use when a camera is not installed. This design enables researchers to record and analyze maternal care quality within the home-cage, allowing flexibility to tailor observations to the specific needs of their experimental design. Additionally, home-cage recordings can detect abnormalities, such as stereotypies, across housing conditions or individual mice, providing further insights relevant to the experimental investigation.

Under normal postpartum conditions, many species including rodents exhibit activation of the HPA axis during and immediately after birth, followed by a steady decrease or hyporesponsiveness of this system during lactation (Douglas et al., 2003; Brunton et al., 2008). Despite our limited sample size (*n* = 3 dams/group), we found a decrease in corticosterone levels in control dams over time; however, corticosterone levels in LBN dams remained elevated throughout the 10 d LBN manipulation, possibly due to their inability to construct a satisfactory nest. Experimental findings using LBN manipulations to examine pup corticosterone, a biomarker of stress, have varied across studies, with reports of LBN-induced increases (Gilles et al., 1996; Avishai-Eliner et al., 2001), decreases (Moussaoui et al., 2016), or no change in pup corticosterone levels (McLaughlin et al., 2016). These variations highlight the importance of standardized LBN parameters across research groups and experiments. In this study, we found that implementing the LBN manipulation from PND 2 to 10 resulted in lower blood serum corticosterone concentrations in LBN pups compared with controls when assessed at PND 10. Low circulating corticosterone levels in LBN pups support the hypothesis that ELS delays HPA axis maturation (Heim et al., 2000; Kaufman et al., 2000; Elzinga et al., 2008; van Bodegom et al., 2017). However, this should be confirmed with a larger sample size and additional timepoints, which is critical for a more comprehensive understanding of these effects.

We found that pups reared under the defined LBN conditions exhibited 18–39% lower total body weight compared with control pups, which is a consistent outcome observed in other low-resource ELS protocols (Rice et al., 2008; Arp et al., 2016). Research groups interested in using this protocol should carefully establish reasonable criteria for pup weight gain to ensure that the LBN procedure does not induce stress in dams and pups that exceeds the institutional research animal welfare standards or significantly increase the risk of pup mortality due to malnourishment. Implementing measures such as adequate temperature control, regular pup weight monitoring, and routine health checks with clear, pre-established humane endpoints can help mitigate the risk of unintentional mortality while still creating a low-resource, stressful environment suitable for safe and ethical biological exploration.

A potential limitation of LBN protocols is that this form of ELS manipulation could act as both a physical low-resource stressor, affecting thermoregulation in pups due to poor nest quality, and a psychological stressor, due to abnormalities in maternal care quality. Consequently, the effects of ELS may reflect the direct effects of a low-resource environment, the indirect influence of maternal stress and altered rearing behaviors, or a combination of converging mechanisms on pup development. Although some studies did not observe alterations in thermoregulation (Bolton et al., 2019), this may vary depending on the specific environmental conditions of individual animal colonies. Moreover, the absence or presence of temperature control, even if limited to PNDs 2–4, can significantly influence the long-term effects of different protocols. By sharing detailed instructions for the modified LBN paradigm, we aim to offer an additional approach to complement existing chronic ELS models. The described protocol therefore offers an accessible, cost-effective, and validated rodent model of low-resource ELS, supporting further exploration of the complex interplay between early life adversities and mood dysregulation.

## Synthesis

Reviewing Editor: Jibran Khokhar, Western University Department of Anatomy and Cell Biology Decisions are customarily a result of the Reviewing Editor and the peer reviewers coming together and discussing their recommendations until a consensus is reached. When revisions are invited, a fact-based synthesis statement explaining their decision and outlining what is needed to prepare a revision will be listed below. The following reviewer(s) agreed to reveal their identity: Nuria Daviu, Marco Bocchio.

The manuscript presents a significant advancement in the field by introducing a standardized, open-source apparatus for inducing early-life stress (ELS) using the LBN paradigm. This tool has the potential to improve reproducibility and comparability of findings across laboratories, addressing the high variability in stress paradigms. Additionally, its low-cost design ensures accessibility for laboratories with varying funding levels, helping to mitigate economic inequalities in scientific research. While this is a valuable step toward standardization, further evidence is needed to clarify the extent to which the apparatus effectively reduces variability across different settings. The reviewers suggested the following areas for improvement:

Major Critiques:

Reduction in Variability Across Labs:

The manuscript should better contextualize the extent of variability in LBN protocols across laboratories. A comparison of published studies highlighting key discrepancies would strengthen this point.

If available, data comparing outcomes from previous LBN setups (e.g., pup weight, survival rates, pup loss) to the proposed apparatus would provide direct evidence of its advantages in standardization and reproducibility.

Statistical Analysis and Sample Size:

The manuscript should consistently report the number of samples and statistical tests used in both the main text and figure legends.

In Figure 2A, the rationale for using linear regression is unclear-would a two-way ANOVA be more appropriate?

Small sample sizes (e.g., n=3) limit statistical power. Increasing sample sizes (e.g., to n=5) would improve robustness and reliability.

Maternal Behavior and Stereotypy Data:

The claim that fragmented maternal behaviors were replicated should be supported with additional data, such as the number and average length of pup visits.

The significant appearance of stereotypic behavior was primarily driven by a single subject, making it difficult to conclude that this method induces stereotypy. A larger sample size is needed to substantiate this claim.

Mortality Rates in LBN Models:

High mortality is a known limitation of LBN procedures. The authors should discuss whether their standardized method mitigates this issue, as it could be a key advantage of their approach.

Minor Critiques:

Clarification of Experimental Timeline:

It is unclear whether the animals are moved to the LBN cage on PND2 or if they are born in it and the mesh is inserted on PND2. This should be explicitly stated.

Assembly Process and Reproducibility Details:

A figure illustrating the cage lid assembly process and an assembly diagram for the mesh component would improve clarity and ensure correct setup.

More precise details on the type and amount of bedding used under the platform would enhance reproducibility.

Temporal Mismatch Between Maternal Care and Corticosterone Levels:

LBN dams spent significantly less time with their pups at PND4, but this effect disappeared by PND6 and PND9. Meanwhile, corticosterone levels were unchanged at PND4 but significantly elevated at PND6 and PND9. A deeper discussion of this discrepancy would be valuable.
