# Supplementary material for: An Open-Source and Highly Adaptable Rodent Limited Bedding and Nesting Apparatus for Chronic Early Life Stress
Source: eNeuro. 2025 Jun 19;12(6):ENEURO.0081-25.2025. doi: 10.1523/ENEURO.0081-25.2025 (PMC12203765; doi:10.1523/ENEURO.0081-25.2025)
Supplement: Figure 2-1 — Extended statistical results supporting Figure 2. Download Figure 2-1, DOC file. [file eneuro-12-ENEURO.0081-25.2025-s002.doc]

**Figure 2-1: Extended statistical results supporting Figure 2**

| **Statistical Test** | **Panel** | **x-axis variable** | **y-axis variable** | | | | **Group**  **(comparison)** | **p-value** | **F stat; df (t stat)** | **R squared**  **(group n)** |
| --- | --- | --- | --- | --- | --- | --- | --- | --- | --- | --- |
| Simple Linear Regression | A | PND | Corticosterone (pg/mL) | | | | LBN | P=0.7664 | *F* (1,7) = 0.09545 | R^2^ = 0.01345 |
|  |  |  |  |  |  |  | CTRL | P=0.0141 | *F* (1,7) = 10.56 | R^2^ = 0.6014 |
| Nonlinear Fit |  |  |  |  |  |  | Comparison of Fits | P=0.0499 | *F* (2,14) = 3.743 |  |
|  | | | | | | | | | | |
| Two-Way ANOVA, Bonferroni's multiple comparisons test | A | PND | Corticosterone (pg/mL) | | | | Interaction | P=0.2117 | *F* (2, 12) = 1.772 |  |
|  |  |  |  | Planned comparison: PND 9-10 | | | | P=0.0484 | t (12) = 2.196 |  |
|  |  |  |  | | | | Time | P=0.1149 | *F* (2, 12) = 2.606 |  |
|  |  |  |  |  |  |  | Group | P=0.1078 | *F* (1, 12) = 3.020 |  |
|  | B | PND | % of time with pups (night) | | | | Interaction | P=0.1754 | F (2, 24) = 1.874 |  |
|  |  |  |  |  |  |  | Time | P=0.3857 | F (2, 24) = 0.9915 |  |
|  |  |  |  |  |  |  | Group | P=0.3012 | F (1, 24) = 1.116 |  |
|  | C | PND | # nest entries (night) | | | | Interaction | P=0.9420 | F (2, 24) = 0.05992 |  |
|  |  |  |  |  |  |  | Time | P=0.8791 | F (2, 24) = 0.1296 |  |
|  |  |  |  |  |  |  | Group | P=0.0005 | F (1, 24) = 16.07 |  |
|  | D | PND | Avg length of nest visits | | | | Interaction | P=0.5969 | F (2, 24) = 0.5273 |  |
|  |  |  |  |  |  |  | Time | P=0.7829 | F (2, 24) = 0.2472 |  |
|  |  |  |  |  |  |  | Group | P=0.0307 | F (1, 24) = 5.269 |  |
|  | G | PND | Weight (g) | | | | Interaction | P<0.0001 | F (8, 315) = 13.48 |  |
|  |  |  | | | PND2: LBN vs. CTRL | | | P=0.0632 | t (315) = 2.714 |  |
|  |  |  |  |  | PND3: LBN vs. CTRL | | | P<0.0001 | t (315) = 5.361 |  |
|  |  |  |  |  | PND4: LBN vs. CTRL | | | P<0.0001 | t (315) = 7.778 |  |
|  |  |  |  |  | PND5: LBN vs. CTRL | | | P<0.0001 | t (315) = 9.101 |  |
|  |  |  |  |  | PND6: LBN vs. CTRL | | | P<0.0001 | t (315) = 11.14 |  |
|  |  |  |  |  | PND7: LBN vs. CTRL | | | P<0.0001 | t (315) = 11.45 |  |
|  |  |  |  |  | PND8: LBN vs. CTRL | | | P<0.0001 | t (315) = 12.35 |  |
|  |  |  |  |  | PND9: LBN vs. CTRL | | | P<0.0001 | t (315) = 13.86 |  |
|  |  |  |  |  | PND10: LBN vs. CTRL | | | P<0.0001 | t (315) = 12.32 |  |
|  |  | PND | Weight (g) | | | Time | | P<0.0001 | F (8, 315) = 223.6 |  |
|  |  |  |  |  |  | Group | | P<0.0001 | F (1, 315) = 823.4 |  |
|  | | | | | | | | | | |
| Mann-Whitney U test | F | Group | Corticosterone (pg/mL) | | | LBN vs. CTRL | | P=0.0044 | U = 78 | LBN(n=17); CTRL(n=20) |
